# Mortality Rate of Ischemic Stroke Patients Undergoing Decompressive Hemicraniectomy With Obesity

**DOI:** 10.7759/cureus.24069

**Published:** 2022-04-12

**Authors:** David R Hallan, Zachary Freedman, Elias Rizk

**Affiliations:** 1 Neurosurgery, Penn State Health Milton S. Hershey Medical Center, Hershey, USA

**Keywords:** craniectomy, obesity paradox, decompression, survival, outcomes, mortality rate, obesity, hemicraniectomy, ischemic stroke, neurosurgery

## Abstract

Background

Obesity has been shown to have a positive mortality benefit in patients undergoing percutaneous coronary intervention and dialysis and those with rheumatoid arthritis, chronic obstructive pulmonary disease, and various wasting diseases. Studies for this mortality benefit in ischemic stroke patients are conflicting and have not been well studied in hemicraniectomy patients. We sought to determine the impact of obesity on outcomes of hemicraniectomy patients.

Methods

We performed a retrospective case-control database analysis using a multi-institutional database (TriNetX) looking at obese versus non-obese patients with ischemic stroke undergoing hemicraniectomy. Our primary endpoint was mortality. Secondary endpoints included seizure, pulmonary embolism, myocardial infarction (MI), cerebral infarction, deep vein thrombosis, tracheostomy, and percutaneous endoscopic gastrostomy. Cohorts were propensity-score matched for confounders.

Results

After propensity score matching for basic demographics and common comorbidities, as well as indicators of stroke severity, 646 patients were identified that were obese and had an ischemic stroke with subsequent hemicraniectomy (cohort 1), and 646 patients were identified who were non-obese with ischemic stroke and hemicraniectomy (cohort 2). Thirty-day survival rate was 98.142% in the obese vs. 87.771% in the non-obese cohorts, 90-day survival was 85.15% vs. 79.35%, 180-day survival was 96.44% vs. 84.52%, 365-day survival was 94.272% vs. 81.734%, and five-year survival was 81.889% vs. 75.077%, respectively. At five years, risk difference was −7.276% (95% CI: −11.757, −2.794) and odds ratio was 0.666 (95% CI: 0.510, 0.871) (p = 0.0029). Despite a higher mortality rate, obese patients had a statistically significant increase in pulmonary embolism (11.61% vs. 5.108, p < 0.0001), deep venous thrombosis (16.873% vs. 9.133%, p < 0.0001), and MI (8.824% vs. 5.882%, p = 0.0428). There was no significant difference in intensive care unit length of stay, ventilator dependence, tracheostomy placement, percutaneous endoscopic gastrostomy placement, or intracerebral hemorrhage.

Conclusions

Despite the increased risk of ischemic stroke, obese patients who undergo hemicraniectomy have decreased mortality rates compared to their non-obese counterparts.

## Introduction

The obesity pandemic has only gotten worse over the years without any end in sight. Numbers estimate that over two billion of the world’s population is considered obese [1]. This pandemic comes with numerous adverse outcomes in those affected including increased risks of various cancers, diabetes, cardiovascular disease, pulmonary disease, musculoskeletal problems, and premature death in addition to a range of stigma, discrimination, bias, and psychological factors that obese individuals experience. Every organ system is affected in obese individuals including the central nervous system (CNS). This effect on the CNS is believed to be from free fatty acid-induced lipotoxicity, oxidative stress, endoplasmic reticulum stress, sympathetic alterations, and metabolic dysfunction [2].

It has been well documented that obesity increases the risk of stroke [3,4]. It has been shown that for each increase in body mass index (BMI), the risk of stroke rises by roughly 6% [4]. However, paradoxically, it has been shown that obesity can provide a positive mortality benefit to these stroke patients although not all studies agree [4-16]. This paradox is known as the obesity paradox and it was first introduced in 2002 by Gruberg et al., who described an increased mortality rate in obese individuals with coronary artery disease [17]. Since then, numerous studies have been published backing the obesity paradox and showing an increased mortality benefit in conditions such as ischemic stroke, kidney disease, pulmonary disease exacerbation, and coronary artery disease [18-20]. The majority of literature outlining and detailing the obesity paradox has been shown through cardiologic, pulmonary, vascular, and renal diseases, but little is known surrounding the obesity paradox and neurosurgical operations, particularly brain operations. Physicians may not see the obesity paradox as a contributing factor in this domain as there is only a small amount of adipose tissue in and around the skull and brain compared to other areas of the body.

Although a recent study looked at mortality as an outcome for ischemic stroke patients undergoing mechanical thrombectomy and found that BMI is associated with decreased mortality, the effects of obesity on ischemic stroke patients undergoing hemicraniectomy are not well studied [21]. This study aims to determine the predictive role of obesity in stroke patients undergoing hemicraniectomy.

## Materials and methods

This is a retrospective case-control study. A de-identified database network (TriNetX) was used to retrospectively query via the International Classification of Diseases, Tenth Revision (ICD-10) and Current Procedural Terminology codes to evaluate all patients with a diagnosis of ischemic stroke and obesity (ICD-10 E66) who subsequently underwent hemicraniectomy (cohort 1) and compared this to ischemic stroke patients without obesity who subsequently underwent hemicraniectomy (cohort 2). Data came from 58 healthcare organizations (HCOs) spanning six countries. This database includes demographic variables, diagnoses, medications, laboratory values, genomics, and procedures. The identity of the HCOs and patients is not disclosed to comply with ethical guidelines against patient re-identification. Because of the database's federated nature, an IRB waiver has been granted. Our use of this database and its validity was disclosed by previous literature, and exact details of the network have been previously described [22]. The index date (date from which time frames were based upon) was set at the date of hemicraniectomy.

The medical information included age at index date, as well as sex, race, and comorbidities such as hypertension, acute kidney injury, diabetes, ischemic heart disease, heart failure, atrial fibrillation, lipoprotein metabolism disorders, dyslipidemia, obesity, history of nicotine dependence, chronic respiratory disease, cirrhosis, alcohol abuse or dependence, and peripheral vascular disease, and was recorded up to the date of the index date. Antiplatelet and anticoagulation medications were likewise recorded. Our primary outcome of interest was mortality, with secondary outcomes of mechanical ventilation, tracheostomy, percutaneous endoscopic gastrostomy (PEG) tube placement, seizure, pulmonary embolism (PE), myocardial infarction (MI), ischemic stroke, and deep venous thrombosis (DVT). These outcomes were followed-up over a period of five years, with interval analysis at 90, 180, and 365 days. Analysis was performed using unmatched and propensity score-matched cohorts using the greedy-nearest neighbor algorithm with a caliper of 0.1 pooled standard deviations. Hazard ratios were calculated. Chi-square analysis was performed on categorical variables. The level of significance was set at a p-value of 0.05.

## Results

A total of 1903 patients were identified with an ischemic stroke diagnosis with obesity and subsequent decompressive hemicraniectomy procedure (cohort 1), while 5530 were included in the non-obese group (cohort 2). A total of 646 patients remained in each group after propensity score matching. After matching, age at diagnosis was 53.79 ± 16.50 and 53.70 ± 19.10 years for cohorts 1 and 2, respectively. A total of 52.167% of patients in cohort 1 and 50.619% in cohort 2 were males. A total of 69.814% vs. 71.672% of patients were white, 21.517% vs. 20.124% were black or African American, and 7.121% vs. 6.037% were of unspecified race. Baseline demographics and characteristics such as diagnosis, acute thrombolytic medication, signs and symptoms, and invasive procedure status are shown in Table 1.

**Table 1:** Baseline demographics and characteristics after propensity score matching Cohort 1: Ischemic stroke diagnosis with obesity and subsequent decompressive hemicraniectomy procedure. Cohort 2: Ischemic stroke diagnosis without obesity and subsequent decompressive hemicraniectomy procedure. ICD-10: International Classification of Diseases, Tenth Revision; CPT: Current Procedural Terminology.

		Before matching			After matching		
Codes (ICD-10, medication, or CPT)	Diagnosis	Cohort 1, n (%)	Cohort 2, n (%)	Std. diff.	Cohort 1, n (%)	Cohort 2, n (%)	Std. diff.
AI	Age at index	55.41 (100.000)	56.26 (100.000)	-	53.79 (100.000)	53.69 (100.000)	-
M	Male	920 (48.884)	3230 (59.605)	0.2165	337 (52.167)	327 (50.619)	0.030975
F	Female	962 (51.116)	2189 (40.395)	0.2165	309 (47.833)	319 (49.381)	0.030975
2106-3	White	1303 (69.235)	3824 (70.567)	0.0290	451 (69.814)	463 (71.672)	0.04084
2054-5	Black or African American	409 (21.732)	910 (16.793)	0.1255	139 (21.517)	130 (20.124)	0.034318
2131-1	Unspecified race	142 (7.545)	564 (10.408)	0.1003	46 (7.121)	39 (6.037)	0.043719
2028-9	Asian	20 (1.063)	101 (1.864)	0.0668	10 (1.548)	13 (2.012)	0.035126
I10-I16	Hypertensive diseases	1476 (78.427)	3268 (60.306)	0.4009	418 (64.706)	402 (62.229)	0.051454
E78	Disorders of lipoprotein metabolism and other lipidemias	982 (52.179)	1828 (33.733)	0.3793	244 (37.771)	237 (36.687)	0.022417
E08-E13	Diabetes mellitus, type 1 and type 2	786 (41.764)	1097 (20.244)	0.4784	181 (28.019)	165 (25.542)	0.055955
R53	Malaise and fatigue	559 (29.702)	1121 (20.686)	0.2088	149 (23.065)	119 (18.421)	0.114722
F17	Nicotine dependence	468 (24.867)	1127 (20.797)	0.0971	123 (19.040)	104 (16.099)	0.077343
I20-I25	Ischemic heart diseases	525 (27.896)	1090 (20.114)	0.1829	120 (18.576)	108 (16.718)	0.048742
R13	Aphagia and dysphagia	550 (29.224)	1295 (23.897)	0.1208	115 (17.802)	108 (16.718)	0.028677
J40-J47	Chronic lower respiratory diseases	481 (25.558)	825 (15.224)	0.2586	107 (16.563)	105 (16.254)	0.00836
N17-N19	Acute kidney failure and chronic kidney disease	501 (26.621)	926 (17.088)	0.2322	95 (14.706)	86 (13.313)	0.040148
R40	Somnolence, stupor, and coma	459 (24.389)	1214 (22.403)	0.0469	82 (12.693)	70 (10.836)	0.057679
I48	Atrial fibrillation and flutter	366 (19.447)	785 (14.486)	0.1325	76 (11.765)	63 (9.752)	0.06498
I50	Heart failure	383 (20.351)	610 (11.257)	0.2513	71 (10.991)	67 (10.372)	0.020048
Z87.891	Personal history of nicotine dependence	348 (18.491)	587 (10.832)	0.2178	65 (10.062)	59 (9.133)	0.031536
R63	Symptoms and signs concerning food and fluid intake	177 (9.405)	343 (6.330)	0.1144	43 (6.656)	42 (6.502)	0.006244
I73	Other peripheral vascular diseases	136 (7.226)	232 (4.281)	0.1267	35 (5.418)	27 (4.180)	0.057963
F10.1	Alcohol abuse	88 (4.676)	310 (5.721)	0.0471	20 (3.096)	16 (2.477)	0.037629
F10.2	Alcohol dependence	65 (3.454)	220 (4.060)	0.0319	14 (2.167)	15 (2.322)	0.01045
K74	Fibrosis and cirrhosis of the liver	33 (1.753)	60 (1.107)	0.0544	<10 (<1.548)	<10 (<1.548)	0
E65-E68	Overweight, obesity, and other hyperalimentation	1245 (66.153)	10 (0.185)	1.963531	<10 (<1.548)	<10 (<1.548)	0
1191	Aspirin	778 (41.339)	1632 (30.116)	0.2358213	215 (33.282)	209 (32.353)	0.019782
11289	Warfarin	172 (9.139)	281 (5.185)	0.1537802	36 (5.573)	33 (5.108)	0.020656
8410	Alteplase	140 (7.439)	296 (5.462)	0.08053	33 (5.108)	27 (4.180)	0.044147
1364430	Apixaban	65 (3.454)	95 (1.753)	0.1069543	<10 (<1.548)	<10 (<1.548)	0
1114195	Rivaroxaban	34 (1.807)	67 (1.236)	0.0465949	<10 (<1.548)	<10 (<1.548)	0
259280	Tenecteplase	<10 (<0.531)	16 (0.295)	0.0368061	<10 (<1.548)	<10 (<1.548)	0
31500	Intubation, endotracheal, emergency procedure	173 (9.192)	529 (9.762)	0.0194479	33 (5.108)	29 (4.489)	0.028973

Thirty-day survival rates were 98.142% in the obese vs. 87.771% in the non-obese cohorts, 90-day survival rates were 85.15% vs. 79.35%, 180-day survival rates were 96.44% vs. 84.52%, 365-day survival rates were 94.272% vs. 81.734%, and five-year survival rates were 81.889% vs. 75.077%. At five years, risk difference was −7.276% (95% CI: −11.757, −2.794) and odds ratio was 0.666 (95% CI: 0.510, 0.871) (p = 0.0029).

**Figure 1:**
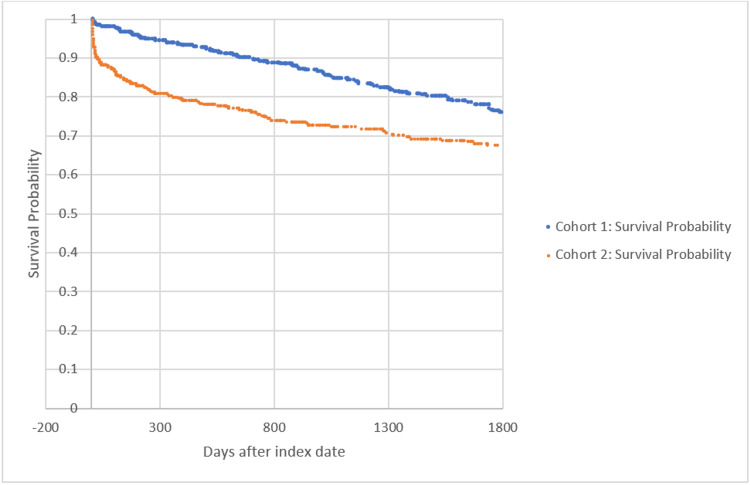
Kaplan-Meier survival analysis for primary outcome: deceased Cohort 1: Ischemic stroke diagnosis with obesity and subsequent decompressive hemicraniectomy procedure. Cohort 2: Ischemic stroke diagnosis without obesity and subsequent decompressive hemicraniectomy procedure.

Figure 1 shows the Kaplan-Meier survival curve for mortality through five years comparing cohorts 1 and 2. The hazard ratio was 0.495, with 95% CI (0.390, 0.628) (p < 0.0001).

Table 2 shows outcomes after propensity score matching. Despite a higher mortality rate, obese patients had a statistically significant increase in PE (11.61% vs. 5.108, p < 0.0001), DVT (16.873% vs. 9.133%, p < 0.0001), and MI (8.824% vs. 5.882%, p = 0.0428). Intensive care unit length of stay was also longer in obese patients at 8.441 ± 10.674 days vs. 7.522 ± 8.110 days, but this was not statistically significant (p = 0.1796). There was no significant difference in ventilator dependence, tracheostomy, PEG, or intracerebral hemorrhage.

**Table 2:** Outcomes after propensity score matching Cohort 1: Ischemic stroke diagnosis with obesity and subsequent decompressive hemicraniectomy procedure. Cohort 2: Ischemic stroke diagnosis without obesity and subsequent decompressive hemicraniectomy procedure.

Outcome	Cohort 1, n (%)	Cohort 2, n (%)	Odds ratio (95% CI)	P-value
Mortality	117 (18.111)	161 (24.923)	0.666 (0.510, 0.871)	0.0029
Ventilator dependence	84 (13.003)	75 (11.610)	1.138 (0.816, 1.587)	0.4459
Tracheostomy	73 (11.300)	69 (10.681)	1.065 (0.752, 1.510)	0.722
Percutaneous endoscopic gastrostomy	87 (13.467)	72 (11.146)	1.241 (0.889, 1.732)	0.204
Pulmonary embolism	75 (11.610)	33 (5.108)	2.44 (1.595, 3.732)	<0.0001
Deep venous thrombosis	109 (16.873)	59 (9.133)	2.019 (1.441, 2.831)	<0.0001
Intracerebral hemorrhage	213 (32.972)	187 (28.947)	1.207 (0.953, 1.529)	0.1177
Myocardial infarction	57 (8.824)	38 (5.882)	1.548 (1.011, 2.370)	0.0428
Intensive care unit length of stay	8.441 ± 10.674 days	7.522 ± 8.110 days	-	0.1796

## Discussion

Unfortunately, the problem of obesity is likely only going to increase [4]. Ever since the concept of the obesity paradox was first introduced, studies have continued to be published detailing the increased mortality benefit in patients with higher BMI in diseases such as heart failure, percutaneous coronary intervention, and dialysis, and those with rheumatoid arthritis, chronic obstructive pulmonary disease, and various wasting diseases [4,23-27]. However, the obesity paradox is still controversial and often literature against the obesity paradox will cite confounding factors associated with the findings. Examples include how BMI is calculated and individuals with a higher muscle mass will have a higher BMI and will be considered obese despite less adipose tissue. Another confounding variable is that most conditions studied have been chronic in nature, which tends to favor older individuals and may not accurately depict the condition [28].

When assessing the obesity paradox in neurological conditions, it has been shown that this paradox exists for conditions such as intracerebral hemorrhage and subarachnoid hemorrhage [29,30]. Researchers found 12 relevant studies that assess stroke and the obesity paradox. Of these 12 studies, 10 have shown higher BMI to be associated with lower mortality [4-14]. Two of them did not show such an association, when adjusted for stroke severity and when looking at mortality only within a 30-day period [15,16]. To our knowledge, this is the first study looking at and approaching a possible outcome correlation between ischemic stroke and obesity in patients' mortality after hemicraniectomy.

Our results demonstrate a significant decrease in mortality in patients with obesity who have undergone decompressive hemicraniectomy after ischemic stroke, as seen in the Kaplan-Meier survival curve in Figure 1, despite an increased rate of PE, DVT, and MI in the obese cohort. This study could be expanded on using prospective longitudinal cohort analysis to assess the validity of the data presented.

Our analysis was not without limitations. The major limitation of this study was that it was retrospective. Furthermore, due to the nature of the database, we were unable to collect patient-level data on specific outcomes. We were unable to report on radiology information. We do not have information on the type of diagnostic test used for confirmation of disease. The BMI is not disclosed, and we do not know the adipose to muscle ratio. In addition, some misidentification is inevitable in database studies.

## Conclusions

Obesity is a health condition that is becoming more prominent. And with increased obesity comes an increased risk of stroke. In this study, we examined the impact of obesity on stroke patients who undergo hemicraniectomy as a life-saving procedure. Our results denote that, paradoxically, obesity is a protective factor against the mortality of this procedure, as compared to an obese patient's non-obese counterpart. Further studies are needed to understand the physiological explanation of these findings, for recommendations to be made concerning patient weight in this patient population.
